# Reproductive adverse events in patients with non‐Hodgkin lymphoma treated with chemotherapeutic regimens including cyclophosphamide, doxorubicin, vincristine, prednisone or CHOP with rituximab: A systematic review and meta‐analysis

**DOI:** 10.1002/pdi3.72

**Published:** 2024-05-24

**Authors:** Rong Han, Jie Zhao, Chengjun Yu, Ling Wang, Long Chen, Yang Hu, Shengde Wu

**Affiliations:** ^1^ Department of Urology Children's Hospital of Chongqing Medical University Chongqing China; ^2^ Chongqing Key Laboratory of Structural BirthDefect and Reconstruction Chongqing China; ^3^ National Clinical Research Center for Child Health and Disorders Chongqing China; ^4^ Chongqing Key Laboratory of Pediatrics Chongqing Chongqing China; ^5^ Ministry of Education Key Laboratory of Child Development and Disorders Chongqing China; ^6^ China International Science and Technology Cooperation Base of Child Development and Critical Disorders Chongqing China

**Keywords:** antineoplastic combined chemotherapy protocols, adverse effects antineoplastic combined chemotherapy protocols, therapeutic use cyclophosphamide, adverse effects humans reproduction, tumor

## Abstract

The regimen of cyclophosphamide, doxorubicin, vincristine, and prednisone (CHOP) or CHOP with rituximab (R‐CHOP) is the first‐line treatment for non‐Hodgkin lymphoma (NHL). NHL patients treated with CHOP/R‐CHOP have a high risk of reproductive adverse events. The aim of this article was to evaluate the reproductive toxicity of regimens and make further suggestions on reproductive protection. We systematically searched with appropriate terms from January 1980 to June 2021 for observational studies in patients treated with CHOP/R‐CHOP, without any language restriction. We conducted meta‐analyses of one‐sample proportions of patients suffering reproductive adverse events after using CHOP/R‐CHOP. In addition, subgroup analyses were performed to determine the effect of sex. Nine articles involving 331 patients were included in the meta‐analysis, and the pooled proportion of reproductive adverse events was computed to be 22.3% (95% confidence interval [CI] 11.4%–33.2%; heterogeneity test *Q* = 65.3; *τ* ^2^ = 0.0231; *I* ^2^ = 87.70%; *p* < 0.001) using the random‐effects model. And, the pooled proportion of male gonadal toxicity was 29.2% (95% CI 11.0%–47.4%; heterogeneity test *Q* = 46.65; *τ* ^2^ = 3.055; *I* ^2^ = 89.3%; *p* < 0.0001). The pooled proportion of female gonadal toxicity was 16.5% (95% CI 8.5%–24.5%; heterogeneity test *Q* = 18.6; *τ* ^2^ = 0.0112; *I* ^2^ = 67.8%; *p* = 0.005). The findings suggest that NHL patients have a relatively high risk of reproductive adverse events after treatment with CHOP/R‐CHOP. Men are more likely to have gonadal damage than women. Evaluation of reproductive function is particularly necessary both before and after treatment. Some reproductive protection strategies implemented for patients who want to preserve their fertility.

## Graphical abstract

The regimen of cyclophosphamide, doxorubicin, vincristine, and prednisone (CHOP) or CHOP with rituximab (R‐CHOP) is the first‐line treatment for non‐Hodgkin lymphoma (NHL). Nine articles involving 331 patients were included in the meta‐analysis, the pooled proportion of reproductive adverse events was 22.3% (95% CI 11.4%–33.2%; *p* < 0.001). The findings suggest that NHL patients have a relatively high risk of reproductive adverse events of CHOP/R‐CHOP. Men are more likely to have gonadal damage than women. Reproductive protection strategies are necessary for patients.

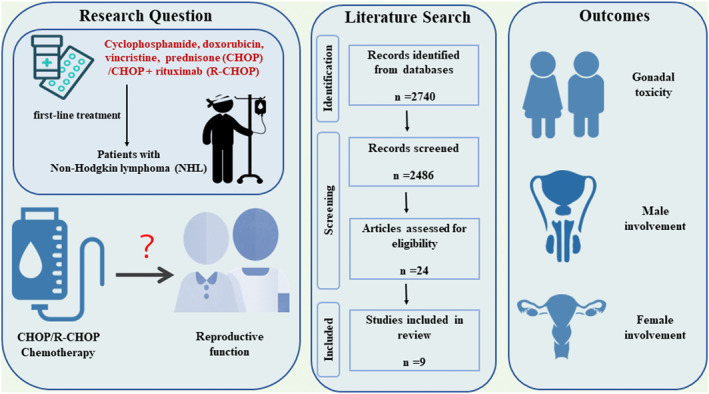

## 1 INTRODUCTION

The preferred chemotherapy treatment for non‐Hodgkin's lymphoma is CHOP^1^(cyclophosphamide, doxorubicin, vincristine, prednisone). CHOP with the addition of rituximab has a better complete response rate and prognosis compared with CHOP alone.^2^ Other adjuvant therapies, such as radiotherapy and neoadjuvant therapy may be added to the treatment regimen for different patients. As the survival rate of patients improves,^3^ patients are increasingly concerned about the side effects associated with chemotherapy, of which the most obvious are myelotoxicity, gonadal toxicity, and cardiovascular toxicity. Both young and old individuals are increasingly attaching more importance to gonadal toxicity caused by treatment. Currently, studies on alkylation agents such as cyclophosphamide show that they have clear gonadal toxicity in humans or animals,^4^, ^5^, ^6^ which is the main basis for the gonadal toxicity of CHOP/R‐CHOP. CHOP‐like chemotherapy plus rituximab versus CHOP‐like chemotherapy alone, results in no significant increase in the frequency of toxicity effects.^7^ At present, many clinical studies have reported that patients receiving CHOP/R‐CHOP chemotherapy may experience temporary or permanent hypofunction of the reproductive system. Once chemotherapeutic drugs are discontinued, the patients’ reproductive function gradually recovers, while a small portion of patients will suffer permanent gonadal damage. Therefore, our systematic review aimed to clarify the statistical significance of the gonadal toxicity of CHOP‐like regimens taken in adult non‐Hodgkin lymphoma (NHL) patients.

## 2 MATERIALS AND METHODS

In this article, we performed a systematic review and meta‐analysis of articles on reproductive toxicity associated with CHOP/R‐CHOP, without any regional or language restriction. In the study, we included NHL patients aged >18 years who were treated with CHOP/R‐CHOP like regimen. We focused on the overall proportion of patients with reproductive adverse events following the use of CHOP/R‐CHOP and measured the proportion of the two subgroups according to sex. Based on the results, we propose a number of methods for reproductive protection.

## 3 SEARCH STRATEGY

This systematic review is reported according to the Preferred Reporting Items for Systematic Reviews and Meta‐analyses Guidelines.^8^ It was registered at the PROSPERO systematic review registration site (registration with PROSPERO: CRD42022333477) with the detailed protocol including the aims and methods of the study.

## 4 DATA ANALYSIS

Study indicators including sample size, patient sex, length of follow‐up, median age, types of drugs, doses of the chemotherapeutic agents, cycles of interval, number of cycles and outcome data, were extracted from the reports as much as possible. If the literature was based on the same clinical trial, we selected one with longer follow‐up and more complete experimental data.

## 5 INFORMATION SOURCES AND SEARCH TERMS

In this study, two independent researchers (RH and JZ) searched for relevant papers using PubMed, the Cochrane Library, and Embase. Articles were systematically searched from January 1, 1980 to June 2021 without language or publication‐type restrictions. In short, the search strategy contained the terms NHL, CHOP/R‐CHOP, gonadal toxicity and some relevant variants of these search terms. Title and abstract screening were conducted by two of us (RH and JZ) in accordance with the predefined criteria. If Hodgkin lymphoma patients and NHL patients were included in an article, we included only reproductive‐related outcomes in NHL patients. In addition, we invited an outstanding expert (Professor Wu) to evaluate the included articles and search for missed articles regarding gonadal effects and CHOP exposure to ensure that our analysis included all relevant studies.

## 6 INCLUSION AND EXCLUSION CRITERIA

In this study, inclusion criteria for selection were: (1) NHL patients aged >18 years; (2) clinical studies or experiments involving regimens that included chemotherapy drugs of cyclophosphamide, adriamycin, vincristine and prednisone; (3) a median duration of remission longer than 2 years; (4) fertility outcomes including parenthood, semen quality, reproductive hormone function (egand. luteinizing hormone, follicle stimulating hormone, testosterone, estrogen), ovarian function, menopausal status and so on. Exclusion criteria for selection were: (1) patients with underlying gonad‐related diseases; (2) studies that include animal and gene; (3) reviews. In addition, the duplicated articles were removed from the list of studies.

## 7 STUDY SELECTION

**FIGURE 1:**
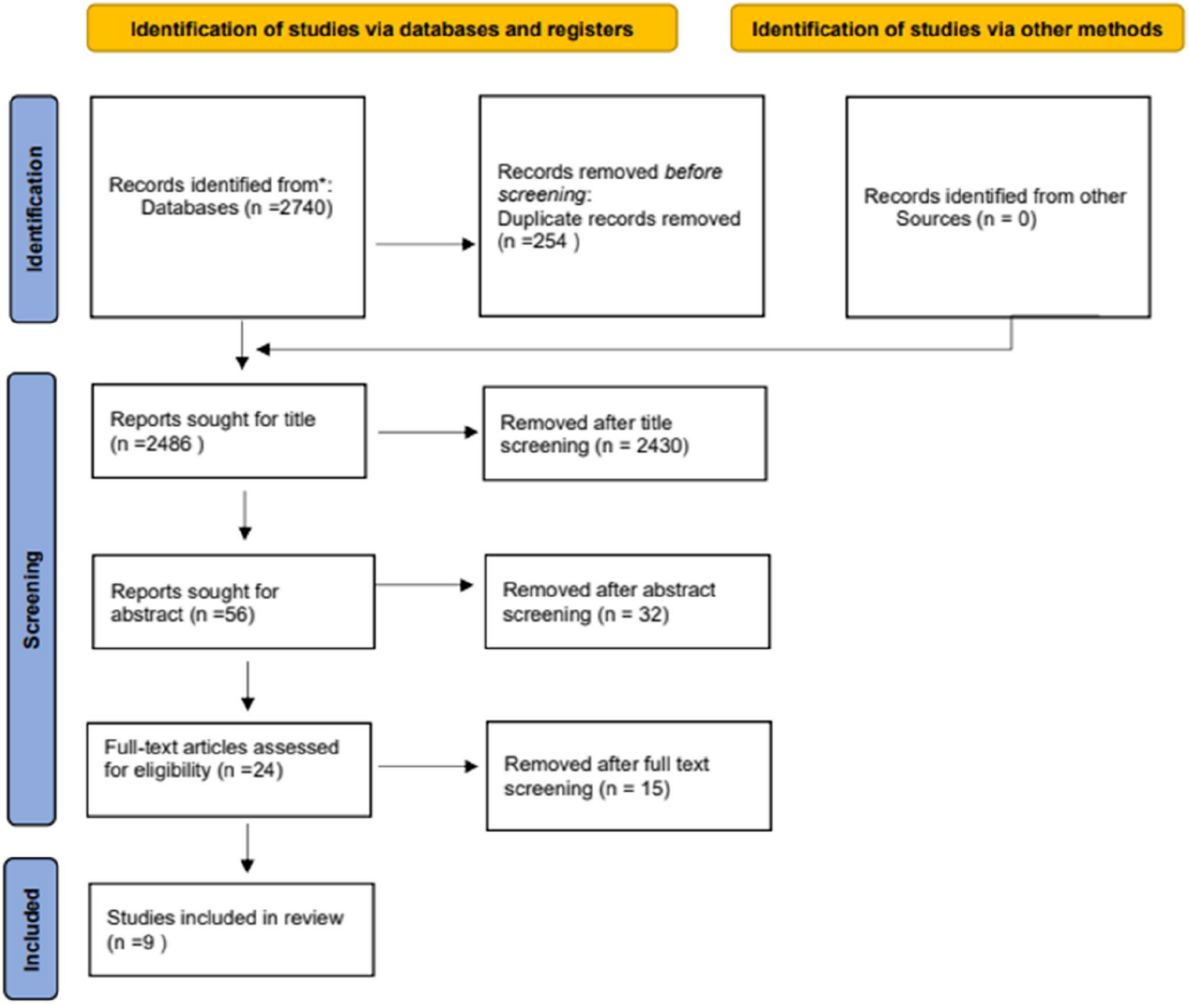
Flow diagram showing studies identified from the literature and their subsequent selection or omission for the meta‐analysis. (*: PubMed, the Cochrane Library, Embas).

The literature search yielded a total of 2740 articles, from which we removed 254 duplicates (Figure 1). Approximately 2430 articles were removed after we screened all titles. After screening the abstracts of 56 articles, we excluded 32 studies. After full‐text assessment, we finally included nine observational studies between 1984 and 2021.^9^, ^10^, ^11^, ^12^, ^13^, ^14^, ^15^, ^16^, ^17^

We were unable to obtain many randomized controlled trials (RCTs) in our searches. Because of the limitations of RCTs, interventions in cancer patients may cause homeopathic exacerbations, which refers to the concept of going beyond adverse effects and being specific to homeopathy. And the adverse effects of treatment are different from homeopathic exacerbations. In this study, we chose to include observational studies.

## 8 DATA EXTRACTION

We carefully evaluated each selected paper and extracted some important information (first author of the study, date of publication, type of study, and study period) and study characteristics (length of follow‐up, sample size, type, and dosage of chemotherapeutic agents).

We extracted the following indicators to represent the functional status of the reproductive system. For male patients, our follow‐up indicators were the patient's hormone level (FSH, LH, testosterone) and sperm‐related parameters (semen volume, sperm count, sperm motility). Similarly, blood hormone levels (FSH, LH, estradiol) and menstrual cycle, pregnancy or fertility were the representative indicators of the ovarian function of female patients. If these indicators are in the range of pathological significance, we consider that patients suffered reproductive adverse events.

Most importantly, the number of total patients and patients with reproductive adverse events after treatment in the selected study was recorded for our systematic review and meta‐analysis (Table 1). Based on gender, we divided the studies into two subgroups. The corresponding number of patients in subgroups was also recorded (Table 2).

**TABLE 1:** Summarized data of papers included in the meta‐analysis.

Study	Number of patients		Proportion^a^
	Reproductive adverse events	Total	
Armitage 1984	12	20	60.00%
Muller 1993	4	30	13.33%
Pryzant 1993	30	58	51.72%
Bokemeyer 1994	4	24	16.67%
Dann 2005	1	13	7.69%
Elis 2006	2	36	5.56%
Meissner 2014	10	36	27.78%
Meissner 2015	8	36	22.22%
Pallotti 2021	6	78	7.69%
Total	77	331	23.26%

^a^ Proportion = (the number of patients with reproductive adverse events/the number of total patients) *100%.

**TABLE 2:** Subgroup data of reproductive included in the meta‐analysis.

Study	Number of male patients		Number of female patients	
	Reproductive adverse events	Total	Reproductive adverse events	Total
Armitage 1984	7	13	5	7
Pryzant 1993	30	58	/	/
Muller 1993	3	19	3	19
Bokemeyer 1994	3	14	1	10
Dann 2005	/	/	1	13
Elis 2006	/	/	2	36
Meissner 2014	7	23	2	13
Meissner 2015	/	/	8	36
Pallotti 2021	6	78	/	/
Total	56	205	22	124

## 9 RISK OF BIAS ASSESSMENT

The methodological quality of the studies included was assessed using the quality assessment tool for observational studies developed by the Joanna Briggs Institute.^18^ Two independent authors (RH and JZ) finished the assessment of the quality of the studies to minimize bias. CJY resolved the discrepancies that arose after two authors had screened full‐text manuscripts through discussion.

## 10 DATA ANALYSIS

We calculated the overall proportion and the corresponding 95% confidence intervals (CIs) provided in the nine manuscripts that were appropriate for our target meta‐analysis. We performed two subgroup analyses for different sexes to evaluate the relationship between gonadal toxicity and sex. In subgroup analyses, we estimated the proportion (95% CI) of patients in different subgroups for reproductive adverse events and overall toxicity. A random‐effects model was used to assess differences in proportions between subgroups. Additionally, if the sample size was large enough, we assessed the effect of each variable on risk (relative risk [95% CI]) for variables such as treatment regimens, the dose of chemotherapeutics, and the number of treatment cycles.

We calculated the heterogeneity of the studies using the *Q* statistic, *I* ^2^, and *τ* ^2^. Heterogeneity was described as low (0%–25%), moderate (25%–50%), large (50%–75%) or considerable (75%–100%).^19^ We applied the fixed‐effects model to pool the total proportions when slight heterogeneity (*I* ^2^ ≤ 50%) was noted; otherwise, a random‐effects model was used.

All statistical calculations were finished in Stata 15 by using the random‐effects model.

## 11 RESULTS

**FIGURE 2:**
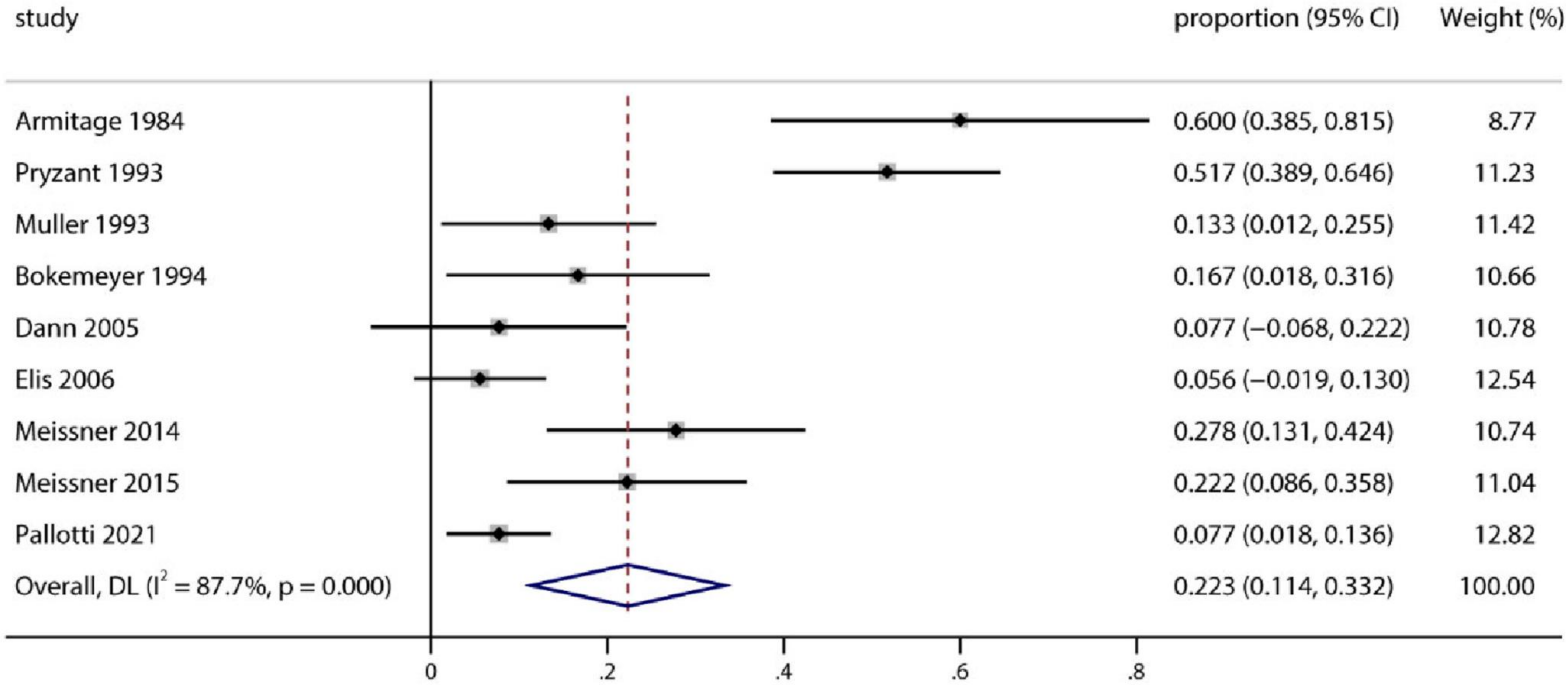
Pooled proportion of patients with reproductive adverse events after treatment.

According to Figure 2, a total of nine observational studies were included in this study. However, we found that not every subgroup sufficiently met the inclusion criteria. Most studies chose CHOP/R‐CHOP plus radiotherapy as the therapeutic regimen for NHL patients. Two papers also utilized the VACOP‐B/MACOP‐B regimen which includes at least cyclophosphamide, doxorubicin, vincristine, and prednisone.^10^ In brief, the articles we included all used these four drugs as basic medical treatment. Nine studies included 377 patients in the meta‐analysis using a random‐effects model (heterogeneity test *Q* = 65.3; *τ* ^2^ = 0.0231; *I* ^2^ = 87.70%; *p* < 0.0001). The pooled proportion of overall gonadal toxicity was calculated as 22.3% (95% CI 11.4%–33.2%).

From Figure 2, we can show that the pooled proportion of overall gonadal toxicity is high. The heterogeneity was 87.70%, which means that the study was highly heterogeneous. According to the forest map, Armitage 1984^9^ and Pryzant 1993,^11^ these two studies with high toxicity rates may be responsible for the high heterogeneity, which may be the reason why the indicators of two articles are not very accurate for the result.

**FIGURE 3:**
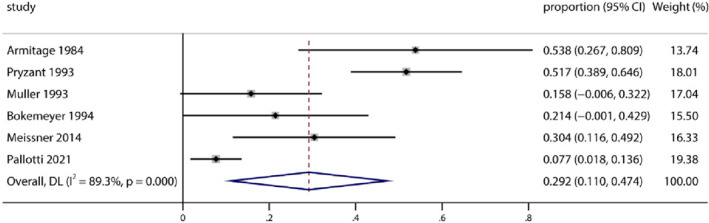
Pooled proportion of male patients with reproductive adverse events after treatment.

**FIGURE 4:**
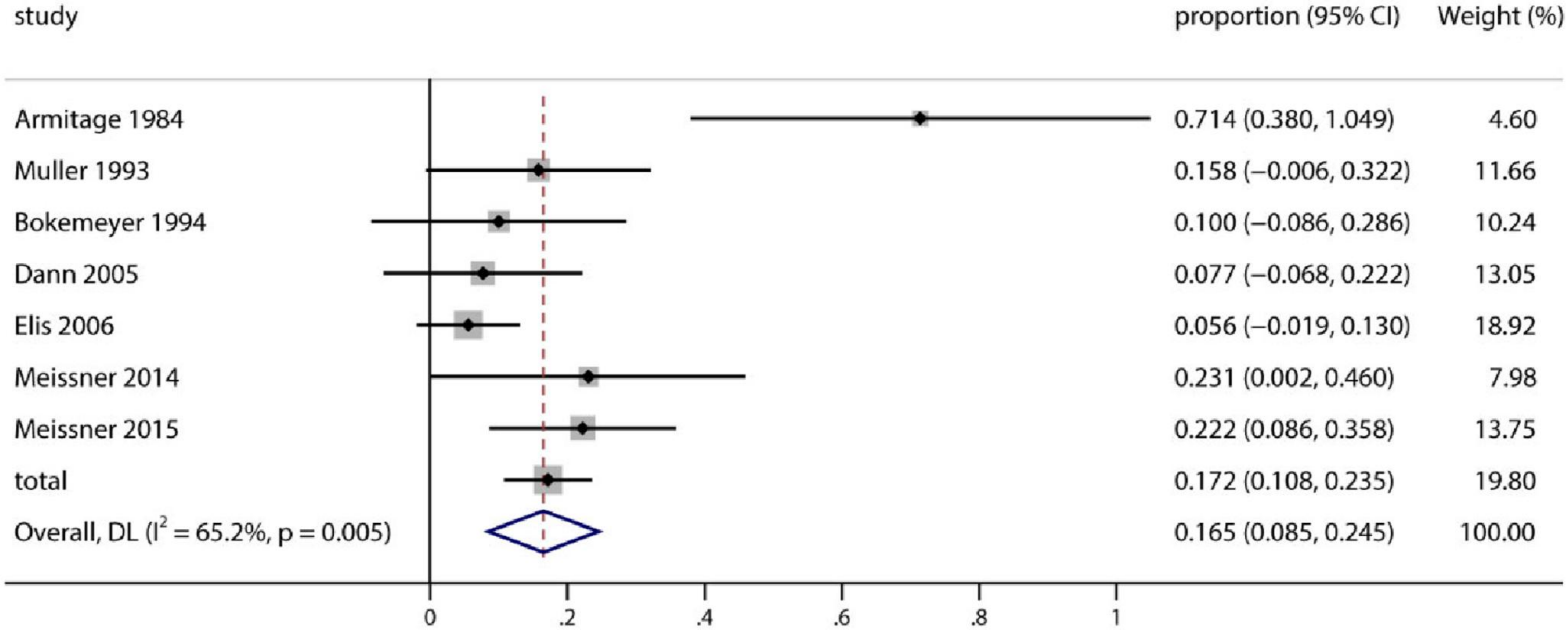
Pooled proportion of female patients with reproductive adverse events after treatment.

At the same time, we performed subgroup meta‐analysis of reproductive toxicity rates in male and female patients. According to Figure 3, 205 male patients were included in this meta‐analysis, which used a random‐effects model (heterogeneity test *Q* = 46.65; *τ* ^2^ = 3.055; *I* ^2^ = 89.3%; *p* < 0.0001). The pooled proportion of male gonadal toxicity was 29.2% (95% CI 11.0%–47.4%). Meanwhile, from Figure 4, 134 female patients were included in the meta‐analysis, which used a random‐effects model (heterogeneity test *Q* = 18.6; *τ* ^2^ = 0.0112; *I* ^2^ = 67.8%; *p* = 0.005), and the pooled proportion of female gonadal toxicity was 16.5% (95% CI 8.5%–24.5%).

From the forest plot, the study Armitage 1984^9^ significantly affected the pooled proportion of gonadal toxicity. Therefore, we used a random‐effects model to reduce heterogeneity.

## 12 DISCUSSION

Overall, our meta‐analysis revealed that for chemotherapy regimens of CHOP/R‐CHOP, the pooled proportion of overall gonadal toxicity was higher (22.3%) than that of other toxicities. Clearly, we can conclude that the proportion of gonadal toxicity of CHOP/R‐CHOP is linked with the sex of the patients. It seems that both male and female patients have a high probability of reproductive adverse events. However, male patients are more likely to suffer from reproductive adverse events than female patients. In these studies, chemotherapy CHOP/R‐CHOP may be the main cause of gonadal toxicity. In this chemotherapy regimen, cyclophosphamide is the main contributor to this side effect of CHOP/R‐CHOP.^20^ As a kind of alkylating agent, cyclophosphamide can cause alkylation of DNA and cross‐links, and is commonly used for antitumor therapy or to reduce transplant rejection.^21^ For men, in the sequence of spermatogenic cells, spermatogonial stem cells and differentiated spermatogonia are easily killed by certain anticancer agents. Spermatogonial stem cells have the ability to self‐renew. DNA damage caused by cyclophosphamide may be repaired at this stage. Cytotoxic drugs may induce low levels of transmissible mutations in spermatogonial stem cells. Spermatocytes, spermatids, testicular sperm and ejaculated sperm are relatively resistant to chemotherapy drugs. Some anticancer drugs, such as cyclophosphamide can induce abnormal chromosome segregation in spermatocytes. Chromosome aberrations and transmissible gene mutations in spermatids and sperm are also induced. Un‐like spermatogonial stem cells, spermatogenic cells at these stages tend to have no ability to deal with DNA damage. A large study indicated that there was no significant increase in birth defects or genetic disease after paternal cytotoxic therapy, with the rate being approximately 4% above that of the general population.^22^, ^23^, ^24^, ^25^, ^26^, ^27^ There are few specific studies on the proportion of birth defects or genetic disease among offspring conceived by patients who receive CHOP/R‐CHOP. One study showed that the cumulative dose of cyclophosphamide was associated with its adverse effects.^28^ As illustrated by this study, treatment with cyclophosphamide at a cumulative dose <4000 mg/m^2^ is unlikely to lead to prolonged azoospermia in male patients. At lower doses, the restoration of sperm motility tends to occur within 3 years, while at higher doses, the damage to the testis can last a long time and can even be permanent. In the article written by Pryzant,^11^ there was a difference in the percentage of patients who recovered to normospermia with different doses of cyclophosphamide. The percentage (83%) of sperm count recovery among patients after receiving <9.5 g/m^2^ of cyclophosphamide and no pelvic radiation was higher than that (47%) of patients who received >9.5 g/m^2^ of cyclophosphamide and no pelvic radiation. Therefore, the higher the cumulative dose of cyclophosphamide, the more likely patients were to suffer reproductive adverse events after treatment.

For women, cyclophosphamide shows a high damage risk to the ovary. It can interfere with cell division via cross‐linking of DNA and induce a decrease in mitochondrial transmembrane potential. As an alkylating agent, cyclophosphamide can inhibit the accumulation of cytochrome C and induce double‐stranded breaks in granulosa cells and oocytes.^21^ Given the difficulty of assessing reproductive function in female patients, more studies are needed to support our conclusions.

Other drugs in CHOP/R‐CHOP can also influence reproductive function.^27^ One study indicated that doxorubicin, an anthracycline antibiotic, can block DNA transcription and replication by intercalation. Ultimately, it induces apoptosis by upregulating the P53 protein and activating ATM. Vincristine alone may not cause damage to the gonads although it can prevent tubulin from forming microtubules. However, doxorubicin and vincristine, two chemotherapy drugs, cause prolonged azoospermia or menopause when added to other cytotoxic drugs, such as cyclophosphamide. There are no studies that prove that prednisone can cause reproductive damage in men or women. Rituximab is not intended to be added to each patient's treatment regimen. The study indicates that rituximab has no gonadal adverse effects. However, unless necessary, pregnant women are not advised to use rituximab because this anti‐CD20 drug can cross the placental barrier and be excreted into breast milk. Transient B‐cell depletion and lymphocytopenia have been observed in children whose mother used rituximab after the first trimester of pregnancy.^29^, ^30^, ^31^

The effect of CHOP/R‐CHOP on reproductive function is a complex process of action that cannot be summarized by a single drug. Moreover, for different genders, the mechanism of action of various drugs is also different, and further study is needed to supplement our conclusions.

We tried to obtain the most accurate data by selecting patients who were treated only with CHOP/R‐CHOP. However, physicians‐in‐charge cannot treat all patients with CHOP/R‐CHOP alone. Depending on the patient's condition and personal willingness, increasing the dose or type of chemotherapy drugs, being combined with radiotherapy, using targeted drugs, or performing hematopoietic stem cell transplantation are considered options for cancer treatment. These treatment options can lead to a higher probability of reproductive damage.^32^ The study revealed that radiation therapy can cause direct gonadal damage, especially abdominal exposure, pelvic exposure, and cranial exposure.^33^ Low‐dose radiotherapy can also disrupt spermatogenesis by inducing ionization of sperm DNA. Another study indicated that the risk of gonadal damage caused by radiotherapy can be augmented when combined with alkylating agents.^34^ However, in 1993, 33 patients treated with radiotherapy alone, including 1 woman and 2 men, received supradiaphragmatic radiotherapy, but none of them experienced reproductive adverse events. Patients treated with chemotherapy alone also did not experience any reproductive adverse events. Three male NHL patients treated with combined therapy suffer gonadal dysfunction. In addition to receiving CHOP, they received abdominal or diaphragmatic radiation and COP maintenance therapy (cyclophosphamide, vincristine, prednisone), their mean cumulative cyclophosphamide dose was 23.3 g, which was 3 times the median cumulative dose of all NHL patients.^11^ We assume that they suffered from gonadal damage caused by an excessive dose of cyclophosphamide.

Meissner in 2015^16^ revealed that 19 patients experienced menopause after treatment, of whom 11 patients resumed periodic menstruation after a short period of time. This result indicated that the damage caused by CHOP/R‐CHOP is not permanent and that reproductive function can return to normal range in many patients. After stopping chemotherapy, reproductive function indicators return to the normal range in many patients. The reproductive system dysfunctions following chemotherapy would be temporary, but organ recovery is unpredictable, and some patients will have severe reproductive damage that cannot be restored.

In the table, the two studies, Armitage in 1984^9^ and Pryzant in 1993,^11^ are the reason why the heterogeneity was >75%. First of all, in Armitage 1984, the final indicator of experimental results was the comparison of patients' sex levels before and after treatment, and we could not obtain the specific degree of the decrease in sex level. Changes in sexual life are related to many factors, of which the individual factors of the participants are considerable. The high median age (59 years) may be one of the reasons for the large number of patients with reproductive toxicity after treatment, because we can learn that 59 years old is not a typical age of fertility and the senility of organs may be mistaken for gonadal toxicity. Second, we see the concrete data of patients in the article written by Pryzant et al. Patients were all men at different stages of the disease. Patients in stages III and IV tend to be treated with dose intensification, pelvic radiation therapy and/or other cytotoxic drugs including methotrexate, etoposide, cytarabine, ifosfamide, and cisplatin. Pryzant assumed that the use of these drugs would not observably alter the restoration of spermatogenesis. Like cisplatin, which is an alkylating agent, has been reported to produce reproductive damage in humans. At the same time, the median age of patients in Pryzant’s report in 1993 was 29 years. Men at this age usually have no primary diseases associated with the reproductive system. However, the pretreatment sperm count of most patients is unknown, and reproductive damage was determined solely by sperm count after treatment. For male patients whose sperm count before treatment was recorded, the significant decrease of the sperm count after treatment can also represent the adverse effect of CHOP/R‐CHOP on reproduction. Finally, the number of patients included in these two studies is too small, so the data are not representative of recent research. We cannot obtain enough data to draw a conclusion about reproductive effects.

Other treatment regimens, such as MACOP‐B (methotrexate, doxorubicin, cyclophosphamide, vincristine, prednisone, bleomycin)/VACOP‐B (cyclophosphamide, adriamycin, oncovin, bleomycin, etoposide, prednisone), CHOP‐B (cyclophosphamide, doxorubicin, vincristine, prednisone, bleomycin), and CHOEP (cyclophosphamide, doxorubicin, vincristine, prednisone, etoposide) all include cyclophosphamide, doxorubicin, vincristine and prednisone. While the dosages of these four drugs may differ from those in the CHOP regimen, they do not have a great influence on the result of gonadal toxicity. Drugs such as methotrexate, bleomycin, and etoposide included in these regimens may temporarily reduce sperm count. They can have additive effects when combined with highly gonadotoxic cyclophosphamide. Also, gonadotropin‐releasing hormone (GnRH) was included in some studies. In a sense, it can function as a preservation treatment at the gonads.

For cancer patients treated with combination therapy, including NHL patients treated with CHOP/R‐CHOP, fertility preservation is an essential protective measure.^35^ If delaying treatment does not greatly affect the patient's outcome, a timely delay of 2–4 weeks can help with fertility preservation. For women with cancer, there are various options for fertility preservation, such as oocyte freezing, embryo freezing, and GnRH treatment. Among them, oocyte freezing and embryo freezing will take approximately 2–4 weeks to complete. Oocyte freezing is suitable for women who do not have a current partner, while embryo freezing, as a more common and successful method, is more suitable for married women or couples. For male patients, methods of fertility preservation are relatively simple and effective, the process of sperm banking takes a short time of 2–4 h. Once bank sperm are completed before cancer treatment, the stored sperm are frozen to complete fertilization via artificial insemination or in vitro fertilization.^36^ For adolescent boys, it is a good choice to pursue testicular tissue cryopreservation at diagnosis to avoid acute adverse events.^37^ Comfortingly, not every patient needs fertility preservation, and most patients need fertility counseling from professionals.^38^ Depalo claimed that cancer patients should carry out fertility preservation as much as possible before treatment in his article. Assisted reproductive treatment was performed in male patients who underwent sperm cryopreservation, and we could notice that thawed sperm motility and vitality had significantly reduced.^39^ The author illustrated that among survivors who wanted to have children, access to frozen semen doubled the odds of becoming fathers after treatment.^40^ There is no denying that regular follow‐up of fertility after treatment is essential for patients who want to preserve their fertility.

In this study, we examined the proportion of gonadal toxicity of CHOP/R‐CHOP and found a significant difference according to sex. Our study has several limitations. First, only 9 studies were included in this meta‐analysis. Although we have used the data we can gather, the sample size is relatively small, and we need more studies to confirm our results further. Second, the treatment of NHL is so complex that the combined treatment regimen may account for high heterogeneity, and we cannot evaluate the toxicity of CHOP/RCHOP alone. More prospective registries are necessary to support our study. Third, we did not compare the results of R‐CHOP/CHOP with those of other regimens, and if possible, we would also assess patients' reproductive function before treatment.

## 13 CONCLUSION

Our results showed that NHL patients treated with CHOP/R‐CHOP are likely to suffer reproductive damage after treatment. At the same time, men are more likely to have adverse reproductive events than women. Therefore, the reproductive toxicity of chemotherapy should be considered in both the men and women of populations of childbearing age. In this study, we also make recommendations to protect reproductive function. Prior to treatment, it is important to assess the patient's reproductive function and perform fertility preservation, such as sperm banking and oocyte freezing, to be performed. Regular follow‐up and further methods, such as using GnRH, are also necessary after treatment. However, more studies and patients are needed to validate our results.

## AUTHOR CONTRIBUTIONS

Rong Han and Shengde Wu conceived and designed the meta‐analysis. Rong Han and Jie Zhao independently searched the Pubmed, Cochrane Library, Embase and independently extracted the data. Rong Han led analysis and interpretation of data, drafted the manuscript and revised content based on feedback. Jie Zhao acted as second reviewer. Chengjun Yu and Jie Zhao assisted with the retrieval of the database and acquisition of data. Ling Wang, Long Chen, and Yang Hu assisted with the interpretation of data and provided critical revision of drafts. Shengde Wu assisted with the conception and design, interpretation of data, and critical revision of drafts. Shengde Wu acted as the corresponding author, provided funding support, assisted with interpretation of data, provided critical revision of drafts and acted as the third (mediating) reviewer.

## CONFLICT OF INTEREST STATEMENT

Shengde Wu is a munber of Pediatric Discovery Editorial Board. To minimaze bias, he was excluded from all the dicision‐making related to the acceptance for publishing. The other authors declared that there are no financial or non‐financial conflicts of interest.

## ETHICS APPROVAL AND CONSENT TO PARTICIPATE

Not applicable.

## CONSENT FOR PUBLICATION

Not applicable.

## Data Availability

All data generated or analyzed during this study are included in this published article and its supplementary information files.
